# Evaluation of the* In Vivo* Therapeutic Effects of Radix Paeoniae Rubra Ethanol Extract with the Hypoglycemic Activities Measured from Multiple Cell-Based Assays

**DOI:** 10.1155/2016/3262790

**Published:** 2016-11-29

**Authors:** Chia-Chuan Chang, Wei Yuan, Yun-Lian Lin, Ren-Shyan Liu, Yi-Chen Juan, Wan-Hua Sun, Huey Jen Tsay, Hsiu-Chen Huang, Yu-Ching Lee, Hui-Kang Liu

**Affiliations:** ^1^School of Pharmacy, College of Medicine, National Taiwan University, Taipei, Taiwan; ^2^Department of Psychiatry, Cheng Hsin General Hospital, Taipei, Taiwan; ^3^Department of Chinese Pharmaceutical Sciences and Chinese Medicine Resources, China Medical University, Taichung, Taiwan; ^4^Department of Nuclear Medicine, Taipei Veterans General Hospital, Taipei, Taiwan; ^5^Institute of Pharmacology, National Yang-Ming University, Taipei, Taiwan; ^6^Department of Life Sciences and Institute of Genome Sciences, National Yang-Ming University, Taipei, Taiwan; ^7^Institute of Neuroscience, Brain Research Center, National Yang-Ming University, Taipei, Taiwan; ^8^Department of Applied Science, National Hsinchu University of Education, Hsinchu, Taiwan; ^9^The Centre of Translational Medicine, Taipei Medical University, Taipei, Taiwan; ^10^The Ph.D. Program for Medical Biotechnology, College of Medical Science and Technology, Taipei Medical University, Taipei, Taiwan; ^11^Division of Basic Chinese Medicine, National Research Institute of Chinese Medicine, Taipei, Taiwan; ^12^Ph.D. Program for the Clinical Drug Discovery from Herbal Medicine, College of Pharmacy, Taipei Medical University, Taipei, Taiwan

## Abstract

*Background*. Radix Paeoniae Rubra (Chi Shao) contains several phytochemicals with hypoglycemic actions. Current research aims to explore potential insulinotropic effects and long-term therapeutic efficacy of such herb against type 2 diabetes.* Methods*. Composition analysis for the ethanol extract (PRExt) was executed by high performance liquid chromatography. Polyphenol-enriched fraction was characterized by high pressure size exclusion chromatography. Multiple cell platforms were employed to evaluate hypoglycemic bioactivities. In animal experiments, blood glucose, the homeostasis model assessment (HOMA)-index assessment, glucose tolerance test, and* in vivo* glucose uptake were all measured. Additional effects of PRExt on obesity and hepatic steatosis were evaluated by serum and histological analysis.* Results*. PRExt provides multiple hypoglycemic effects including the enhancement of glucose-mediated insulin secretion. Pentagalloylglucose and polyphenol-enriched fraction are two insulinotropic constituents. Moreover, PRExt intraperitoneal injection causes acute hypoglycemic effects on fasted db/db mice. Oral administration of PRExt (200 mg/kg b.w.) gradually reduces blood glucose in db/db mice to the level similar to that in C57J/B6 mice after 30 days. The improvement of glucose intolerance, HOMA-index, and* in vivo* glucose uptake is evident in addition to the weight loss effect and attenuation of hepatic steatosis.* Conclusion*. PRExt is an effective antidiabetic herbal extract with multiple hypoglycemic bioactivities.

## 1. Introduction

Diabetes mellitus (DM) is a major health issue, affecting more than 180 million people worldwide. Type 2 diabetes (T2D) affects more people than does type 1 diabetes and the typical comorbidities are central obesity and fatty liver [1]. Hyperglycemia in T2D patients is the consequence of both insulin resistance and beta-cell dysfunction or failure [2]. Therefore, an effective antidiabetic agent should both improve insulin sensitivity and enhance beta-cell function. Herbal medicine containing multiple hypoglycemic constituents has a great potential for fulfilling such requirement.

Radix Paeoniae Rubra (Chi Shao) is prescribed in Traditional Chinese Medicine (TCM) to enhance blood circulation and eliminate blood stasis and is usually employed for treating diabetics, for instance, Xue-Fu-Zhu-Yu decoction and Keishi-Bukuryo-Gan [3, 4]. Recently, we demonstrated that an ethanol extract of Radix Paeoniae Rubra could suppress transcription of the hepatic phosphoenolpyruvate carboxykinase (PEPCK) gene [5].

It was identified that the insulin sensitizing effect of chemical constituents from Radix Paeoniae Rubra is modulated by the inhibition of protein-tyrosine phosphatase 1B [6]. Pentagalloylglucose, another Radix Paeoniae Rubra constituent, acts like an insulin mimetic by binding to the insulin receptor [2, 7]. Additionally, pentagalloylglucose and tannin acids inhibit adipogenesis and counteract obesity [8–10]. Several pharmacological effects of paeoniflorin have been demonstrated, including hypoglycemic, hypolipidemic, and antifatty liver activities [5, 7, 11–14]. Finally, paeonol inhibits intestinal glucose absorption and provides antioxidant effect [15, 16].

Despite the well-known hypoglycemic, antiadipogenesis,, and antiobesity effects of constituents from Radix Paeoniae Rubra, the glucose-mediated insulinotropic effect of Radix Paeoniae Rubra extract and constituents on insulin secreting cells remained to be evaluated. On the other hand, the long-term therapeutic effects of Radix Paeoniae Rubra extract in the context of T2D remain unclear. Therefore, we intend to answer these questions by testing the efficacy of Radix Paeoniae Rubra extract and/or constituents in cell-based platforms as well as in a mouse model of T2D.

## 2. Methods

### 2.1. Preparation of the Ethanol Extract of Radix Paeoniae Rubra (Chi Shao)

The ethanol extract of Radix Paeoniae Rubra (PRExt) was prepared as previously described [5] with some modifications. Briefly, 2 kg of the whole dried root from* Paeonia lactiflora Pall*. was extracted by 80% ethanol (EtOH) overnight. For chemical profiling of the extract, a high performance liquid chromatography (HPLC) apparatus (Shimadzu, Japan) comprising a LC-10AT pump, a SPD-10AV UV-visible spectrophotometric detector, and a Cosmosil 5C18-AR-II (4.6 mm × 250 mm) column was used. The mobile phase was set as follows: solvent A, 0.1% formic acid in H_2_O; solvent B, 0.1% formic acid in acetonitrile; 0–10 min, B = 2–5%; 10–20 min, B = 5–12%; 20–32 min, B = 12–30%; 32–36 min, B = 20–30%; 36–50 min, B = 30–50%; 50–60 min, B = 50–100%. The flow rate was set to 1 mL/min. The preparation of polyphenol-enriched fraction (PEF) was previously described [7]. High Pressure Size Exclusion Chromatography (HPSEC) profiling of PEF was carried out on a size exclusion column (Bio-Gel G3000_XL_).

### 2.2. Cell Culture

The rat hepatoma cell line H4IIE cells were cultured in Dulbecco's Modified Eagle's Medium (DMEM) containing 1 g/L glucose, 5% (v/v) fetal bovine serum (FBS), and 1% (v/v) antibiotics (100 U/mL penicillin and 0.1 g/L streptomycin) [7]. Rat insulin secreting cell line, BRIN-BD11 cells, were routinely cultured with RPMI-1640 containing 2 g/L glucose, 10 mM HEPES, and 1.0 mM sodium pyruvate and supplemented with 10% (v/v) FBS and 1% (v/v) antibiotics [17]. In terms of L6 rat skeletal muscle cell, undifferentiated L6 myoblast was cultured in DMEM containing 1 g/L glucose, 10% (v/v) FBS, and 1% antibiotic/antimycotic solution at a low density. Cells were discarded after being passaged over 8 generations. For myotube differentiation, L6 myoblast was seeded into 12-well plates at a density of 1 × 10^5^ cells/well in DMEM, 2% (v/v) FBS, and 1% antibiotic/antimycotic solution. Medium was replaced every three days and matured myotubes were formed 7 days after seeding [18]. For routing culture, cell lines were maintained at 37°C in an atmosphere of 5% CO_2_ and 95% air.

### 2.3. Reverse-Transcription PCR

The measurement of PEPCK and beta-actin mRNA was previously described [19]. In short, total RNA extracted from treated H4IIE cells was reverse-transcribed. Then, 50 ng of cDNA was used in a PCR reaction to amplify the beta-actin or PEPCK genes. In terms of primer sequences, annealing temperature was used for PCR reaction, beta-actin: 5′-CGTAAAGACCTCTATGCCAA-3′ and 5′-AGCCATGCCAAATGTGTCAT-3′/57°C/349 bp; PEPCK: 5′-AAGGCCGCACCATGTATGTC-3′ and 5′-AGCAGTGAGTTCCCACCGTAT-3′/57°C/319 bp. Finally, PCR products were quantified using GeneTools 3.06 (Syngene, Frederick, USA).

### 2.4. Glucose Uptake Assay

A 2-(N-(7-Nitrobenz-2-oxa-1,3-diazol-4-yl)Amino)-2-deoxyglucose (2-NBDG) uptake assay was carried out based on a previously published protocol [20]. Differentiated L6 myotubes were serum-starved 4 hours prior to exposure to each constituent for 30 minutes at 37°C. Cells were then incubated with 2-NBDG (100 *μ*M) in Krebs-Ringer Bicarbonate Buffer (KRBB) for 1 hour at 37°C. Finally, the fluorescence intensity of cell lysate was quantified using an M5 spectrophotometer (Molecular Devices, CA, USA) using an excitation/emission wavelength of 465/540 nm and normalized by protein concentration of each lysate.

### 2.5. Acute Insulin Secretion Test

The acute insulin secretion test has been previously described [21]. Briefly, seeded BRIN-BD11 cells were preincubated with KRBB (1.1 mM glucose) for 40 minutes at 37°C. Cells were incubated with each constituent for 20 minutes at 37°C. Test medium was then collected and insulin levels in the medium were measured using an Homogeneous Time Resolved Fluorescence (HTRF) insulin assay (Cisbio, USA).

### 2.6. Animal Husbandry and* In Vivo* Evaluation of Hypoglycemic Activity

The use of male C57J/B6 mice and db/db mice (obtained from the National Laboratory Animal Center, Tainan, Taiwan) was approved by the Animal Research Committee at the National Research Institute of Chinese Medicine. All procedures were in accordance with* The Guide for the Care and Use of Laboratory Animals* (NIH publication, 85-23, revised 1996) and the Guidelines of the Animal Welfare Act.

C57J/B6 mice received standard chow and db/db mice were fed high-fat chow (high-fat diet) made from standard chow supplemented with extra lard (20% w/w), cholesterol (1% w/w), and cholic acid (0.1% w/w). For evaluation of acute hypoglycemic effect of PRExt, fasted db/db mice with blood sugars > 500 mg/dL were randomly grouped and anesthetized by intraperitoneal injection with pentobarbital (30 mg/kg). Once initial blood sugar was measured from a cut tail tip using a commercial glucometer (Bioptik Technology, Taiwan), mice received either the vehicle (saline) or PRExt via intraperitoneal injection. Blood glucose was measured within the next 12 hours. In terms of long-term administration, experimental procedures began when the animals were 10 weeks old (the age at which fasting blood glucose in db/db mice rose above 400 mg/dL). During the course of the experiment, db/db mice received vehicle (dH_2_O) or PRExt (200 mg/kg) daily. C57J/B6 mice received vehicle (dH_2_O) only. Treatment was administrated via oral gavage using a stomach tube. Blood sugar was measured from a cut tail tip every 3 days in total of 30 days.

### 2.7. Intravenous Glucose Tolerance Test (IVGTT)

To perform an intravenous glucose tolerance test, mice were fasted overnight and then administered a glucose solution (1 g/kg body weight) via the tail vein. Animals were anesthetized by pentobarbital (30 mg/kg of body weight, i.p.) for these procedures. Blood sugar was measured at 0, 15, 30, 60, and 120 minutes after the glucose was administered.

### 2.8. Small-Animal Positron Emission Tomography (PET) Scanning

The ability of the mice to absorb glucose was evaluated using micro-PET scanning [22]. Briefly, mice were first anesthetized with isoflurane (2% in 100% oxygen). A total of 100 *μ*Ci [^18^F] 2-fluorodeoxyglucose (FDG) dissolved in saline was then injected into each animal through the tail vein. After 1 hour, each mouse was placed on a Concorde micro-PET R4 scanner (Concorde Microsystems, Knoxville, TN) for radioactivity scanning. The region of interest (ROI) in the skeletal muscle was selected, and a cylinder calibration method was used to convert the units of the micro-PET images from counts per second (cps) per voxel (cps/voxel) to nCi per cc (nCi/cc). The results were further normalized to the blood sugar level of each animal and presented as uptake radioactivity (nCi/cc)/serum glucose (mg/dL).

### 2.9. Serum Biochemical Analysis

Serum triglycerides were measured using a FUJI DRI-CHEM 3000 (Fuji Photo Film Co., Tokyo, Japan). Serum insulin concentrations were quantified using a Luminex multiplex assay (cat#: MENDO-75K-05, Millipore Corporation, Bedford, MA). The homeostasis model assessment for insulin resistance (HOMA-IR = fasting blood glucose [mM] × fasting insulin [*μ*U/mL]/22.5) and the homeostasis model assessment for beta-cell function (HOMA-B = 20 × fasting insulin [*μ*U/mL]/fasting blood glucose [mM] − 3.5) were then assessed [23].

### 2.10. Histopathological Examinations

A portion of liver was fixed with 4% (w/v) paraformaldehyde and slides made from paraffin-embedded tissue were used for hematoxylin and eosin (H&E) and Periodic Acid-Schiff (PAS) staining to visualize tissue morphology and determine the glycogen content, respectively. Additionally, the slides of frozen tissue sections were stained with Oil red O (ORO) to quantify neutral lipid and fatty acid contents [23].

### 2.11. Statistics

Significant differences between groups were determined using a Student's *t*-test (Mann–Whitney test) or a one-way analysis of variance (ANOVA) with a Tukey-Kramer test. Results were presented as the mean ± SEM. Differences were considered significant if *p* < 0.05.

## 3. Results

### 3.1. Chemical Analysis of Four Radix Paeoniae Rubra Constituents in Radix Paeoniae Rubra Extract (PRExt)

**Figure 1:**
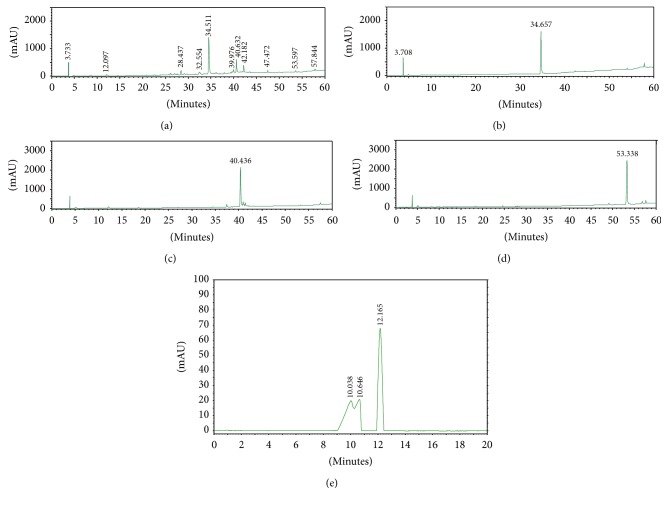
Chromatography profiles of PRExt and chemical constituents. Representative HPLC and HPSEC profiles of (a) PRExt, (b) paeoniflorin, (c) pentagalloylglucose, (d) paeonol, and (e) polyphenol-enriched fraction (PEF).

Representative HPLC and HPSEC profiles of Radix Paeoniae Rubra extract (PRExt) and reference compounds are shown in Figure 1. HPLC peaks representing PRExt constituents, paeoniflorin (retention time = 34.657 min), pentagalloylglucose (retention time = 40.436 min), and paeonol (retention time = 53.338 min) are identified in PRExt. The Lambda max (*λ*max) information for all the chromatograms was listed as below: PRExt: UV *λ*max (MeOH, log⁡*ε*) 230 (3.08), 273 (2.19) nm; paeoniflorin: UV *λ*max (MeOH, log⁡*ε*) 230 (2.43), 276 (0.22) nm; pentagalloylglucose: UV *λ*max (MeOH, log⁡*ε*) 228 (2.50), 267 (1.80), 294 (1.91) nm; paeonol: UV *λ*max (MeOH, log⁡*ε*) 232 (2.50), 311 (2.08) nm; PEF: UV *λ*max (MeOH, log⁡*ε*) 234 (2.53), 312 (2.12), 356 (1.93) nm. The yield of fractions and compounds: paeoniflorin: 56 mg/g, pentagalloylglucose: 20.9 mg/g, paeonol: 0.68 mg/g, polyphenol-enriched fraction (PEF): <66 mg/g.

Both peaks of PRExt representing paeoniflorin and pentagalloylglucose were further confirmed with mass spectrometry in positive and negative mode, respectively (see Supplementary Figures 1 and 2 of the Supplementary Material available online at http://dx.doi.org/10.1155/2016/3262790). Due to the chemical properties of polyphenol-enriched fraction as macromolecules, chemical profile analysis was performed by employing HPSEC. As shown in Figure 1(e), two major HPSEC peaks are observed and appeared at retention times of 10 and 12 minutes. Estimated molecular weights of these peaks are 73.479 and 13.693 kDa, respectively.

### 3.2. Pharmacological Evaluation of the Hypoglycemic Actions of PRExt and the Four Constituents

**Figure 2:**
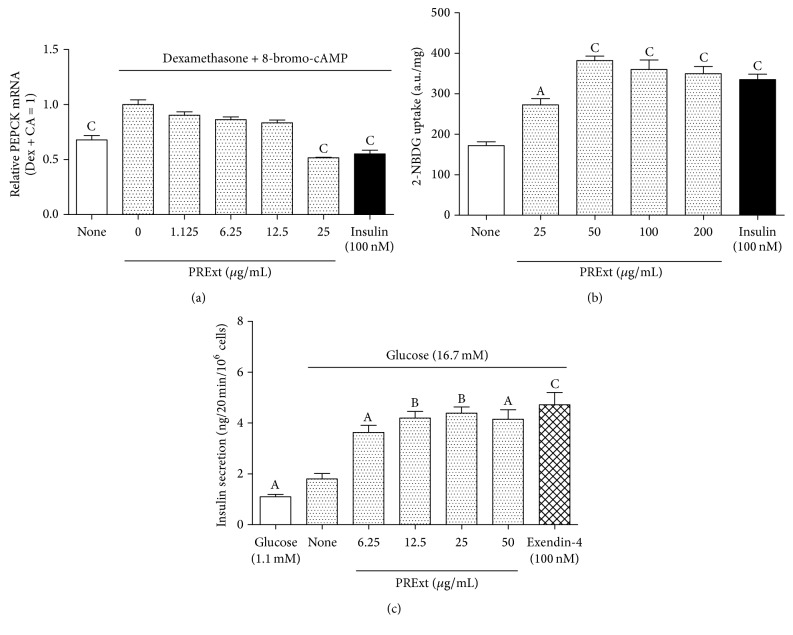
Multiple hypoglycemic activities of PRExt. (a) A dose-dependent suppressive effect of PRExt on PEPCK mRNA expression. Insulin (100 nM) served as a reference drug. Data are presented as the mean ± SEM (*n* = 3/group). ^C^ *p* < 0.001 when compared with mRNA levels in H4IIE cells treated with dexamethasone and 8-bromo-cAMP. (b) The glucose uptake activity of L6 myotubes exposed to PRExt or insulin (100 nM) for 30 minutes prior to performing a 2-NBDG (100 *μ*M) uptake assay. Data are presented as the mean ± SEM (*n* = 6/group). ^A^ *p* < 0.05 and ^C^ *p* < 0.001 when compared with untreated L6 myotubes. (c) The insulinotropic effect of PRExt on BRIN-BD11 cells under hyperglycemic condition (16.7 mM glucose). Exendin-4 (100 nM) was used as a reference drug. Data are presented as the mean ± SEM (*n* = 4/group). ^A^ *p* < 0.05, ^B^ *p* < 0.01, and ^C^ *p* < 0.001 when compared with insulin levels under hyperglycemic conditions (16.7 mM glucose).

We compared the pharmacological activities of the herbal extract (PRExt) and the four constituents of PRExt (paeoniflorin, pentagalloylglucose, paeonol, and PEF) using three cells lines (liver, muscle, and pancreatic beta cells). In the liver cell line (H4IIE), dexamethasone (500 nM) and 8-bromo-cAMP (100 *μ*M) induced PEPCK transcription, and insulin (10 nM) played a dominant role in suppressing this upregulation (*p* < 0.001) (Figure 2(a)). Additionally, PRExt (25 *μ*g/mL) suppressed PEPCK mRNA expression (*p* < 0.001). Next, we performed a glucose uptake assay in differentiated L6 myotubes. As shown in Figure 2(b), 2-NBDG uptake by L6 muscle cells significantly increased after exposure to insulin (100 nM) for 30 minutes (*p* < 0.001). Treatment with PRExt (50 *μ*g/mL) significantly stimulated glucose uptake after 30 minutes (*p* < 0.01).

**Figure 3:**
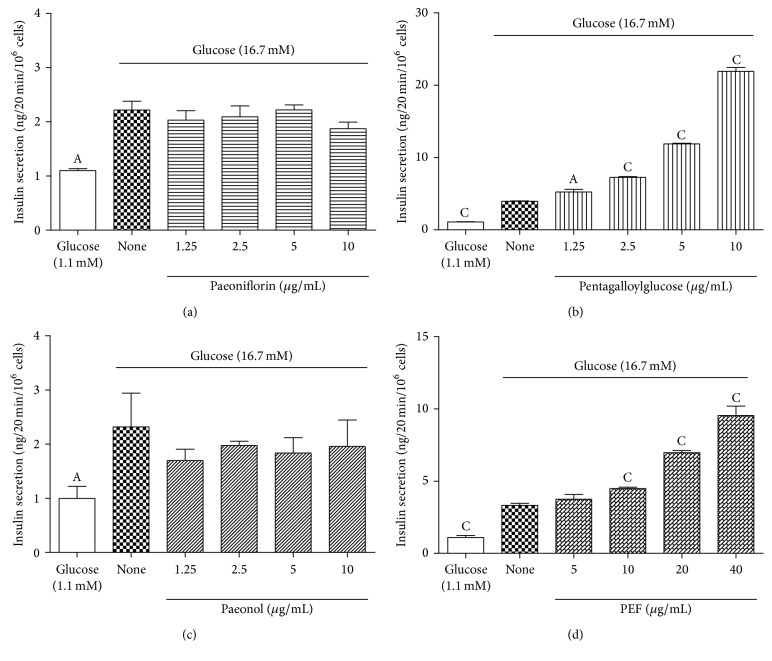
Insulinotropic effects of four constituents of Radix Paeoniae Rubra on glucose-stimulated insulin secretion of BRIN-BD11 cells. The insulinotropic effects of (a) paeoniflorin, (b) pentagalloylglucose, (c) paeonol, and (d) PEF on BRIN-BD11 cells under hyperglycemic conditions (16.7 mM glucose). Data are presented as the mean ± SEM (*n* = 4–8/group). ^A^ *p* < 0.05 and ^C^ *p* < 0.001 when compared with insulin levels under hyperglycemic conditions (16.7 mM glucose).

Finally, we evaluated the acute insulinotropic effects of PRExt and the four constituents on BRIN-BD11 pancreatic islet cells exposed to 16.7 mM glucose. As shown in Figure 2(c), PRExt enhanced insulin secretion by these in a dose-dependent manner. Significant insulinotropic effects induced by PRExt and exendin-4 were observed at a concentration of 6.25 *μ*g/mL (*p* < 0.05) and 100 nM, respectively. In terms of the four constituents, only pentagalloylglucose and PEF enhanced insulin secretion (Figures 3(b) and 3(d)). The increase was significant at a concentration of pentagalloylglucose and PEF at 2.5 *μ*g/mL (*p* < 0.01) and 10 *μ*g/mL (*p* < 0.001), respectively.

### 3.3. PRExt Administration Improves Glycemic Control in db/db Mice

**Figure 4:**
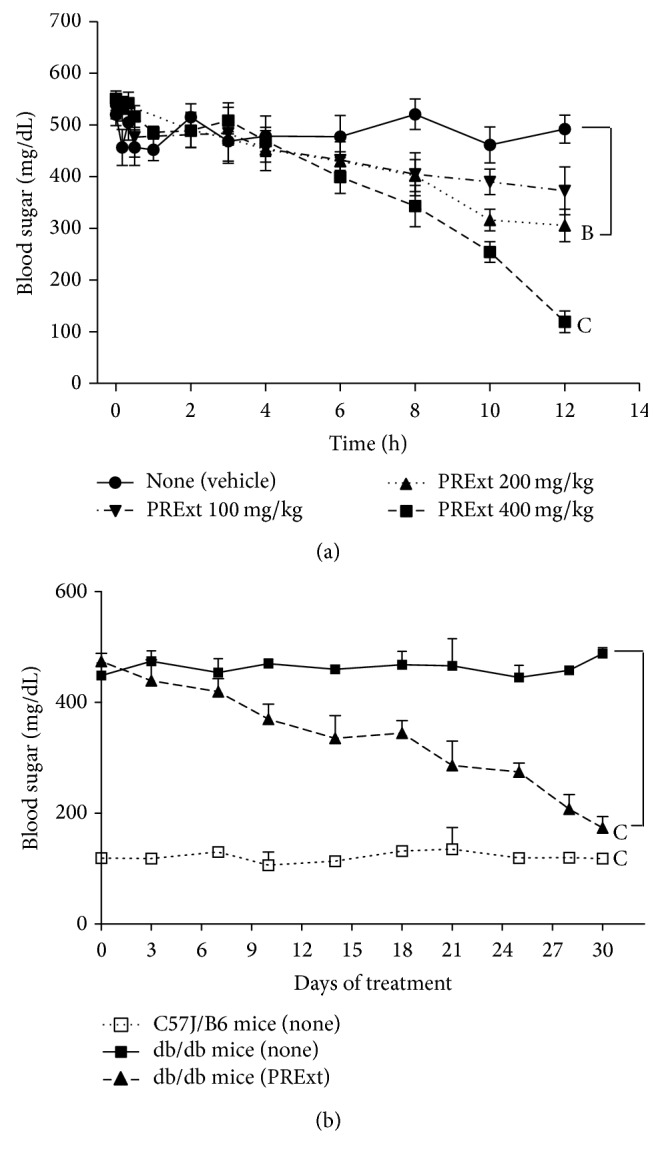
Evaluation acute and long-term hypoglycemic effects of PRExt on db/db T2D mice. (a) Fasting blood glucose of db/db mice were intraperitoneal injected with PRExt at indicated dosages. Acute blood glucose changes are recorded at 0, 15 min, 30 min, 1 h, 2 h, 4 h, 6 h, 8 h, 10 h, and 12 h, respectively. Data are presented as the mean ± SEM (*n* = 5/group). ^B^ *p* < 0.01 and ^C^ *p* < 0.001 when compared with data of vehicle group at 12 h. (b) The long-term state blood sugar of C57J mice and db/db control mice in the presence or absence of oral administration of PRExt (200 mg/kg/day). Data are presented as the mean ± SEM (*n* = 6–10/group). ^C^ *p* < 0.001 when compared with untreated db/db mice.

We found a dose-dependent inhibition of fasting blood sugar in db/db mice treated with PRExt at doses of 200 mg/kg and higher (Figure 4(a)). As a result, we administered PRExt at 200 mg/kg to db/db mice to evaluate its long-term therapeutic efficacy. As shown in Figure 4(b), untreated db/db mice experienced sustained hyperglycemia over 30 days, with a state blood sugar significantly higher than that of untreated C57J/B6 mice (*p* < 0.001). When db/db mice were treated with PRExt (200 mg/kg) for 30 days, the elevated blood sugar gradually decreased and after 30 days was significantly reduced when compared with untreated mice (*p* < 0.001).

**Figure 5:**
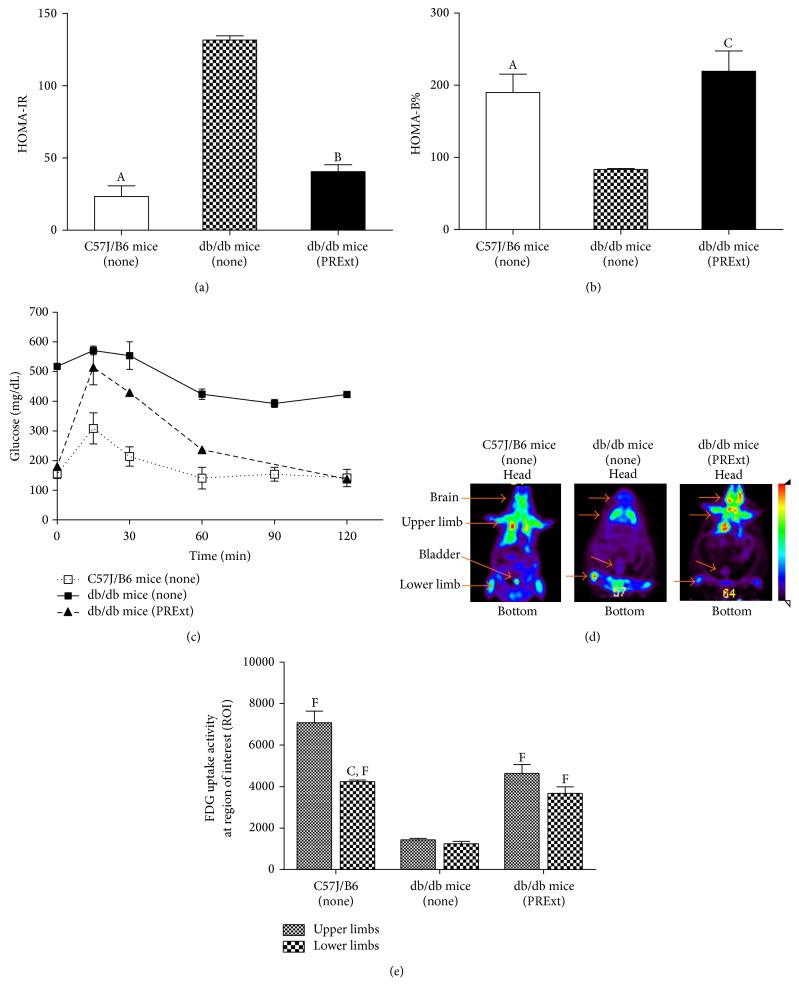
Therapeutic effects of PRExt in treating dysglycemia in db/db T2D mice. The results of (a) HOMA-IR, (b) HOMA-B%, and (c) glucose tolerance of C57J mice and db/db control mice in the presence or absence of PRExt treatment. Data are presented as the mean ± SEM (*n* = 6–10/group). ^A^ *p* < 0.01, ^B^ *p* < 0.05, and ^C^ *p* < 0.001 when compared with untreated db/db mice. (d) Representative results of 2-[^18^F]-fluorodeoxyglucose (FDG) biodistribution measured by micro-PET scanning. (e) Quantitative results of upper and lower limbs after FDG uptake. Data are presented as the mean ± SEM (*n* = 4/group). ^C^ *p* < 0.001 when compared with FDG uptake activity between upper and lower limbs in the same mice. ^F^ *p* < 0.001 when compared with FDG uptake activity of the corresponding limb region in untreated db/db mice.

As shown in Figure 5(a), there was a nearly threefold increase in HOMA-IR in db/db mice compared with C57J mice (*p* < 0.05), and PRExt treatment significantly reduced this HOMA-IR in db/db mice (*p* < 0.01). As illustrated in Figure 5(b), a twofold reduction in HOMA-B% was observed in db/db mice compared to C57J mice (*p* < 0.05), but HOMA-B% in db/db mice was more comparable to that of C57J mice after treatment with PRExt (*p* < 0.001). In Figure 5(c), untreated db/db mice were obviously glucose intolerant; in contrast, glucose intolerance clearly improved in mice treated with PRExt. Finally, as illustrated in Figure 5(d), a one-hour ^18^FDG PET scan of the brain, limbs, heart, and bladder revealed poor glucose absorption and biodistribution in the db/db mice compared to C57J/B6 mice. However, upon PRExt treatment, glucose biodistribution clearly improved. As seen in Figure 5(e), glucose absorption was higher in the upper limbs than in the lower limbs of C57J/B6 mice (*p* < 0.001), but there was no difference in glucose absorption between the limbs of db/db mice, whether they had been treated with PRExt or not. However, there was a twofold increase in glucose within individual limbs of db/db mice upon PRExt treatment (*p* < 0.001).

### 3.4. PRExt Administration Ameliorates Both Obesity and Hepatic Steatosis in db/db Mice

**Figure 6:**
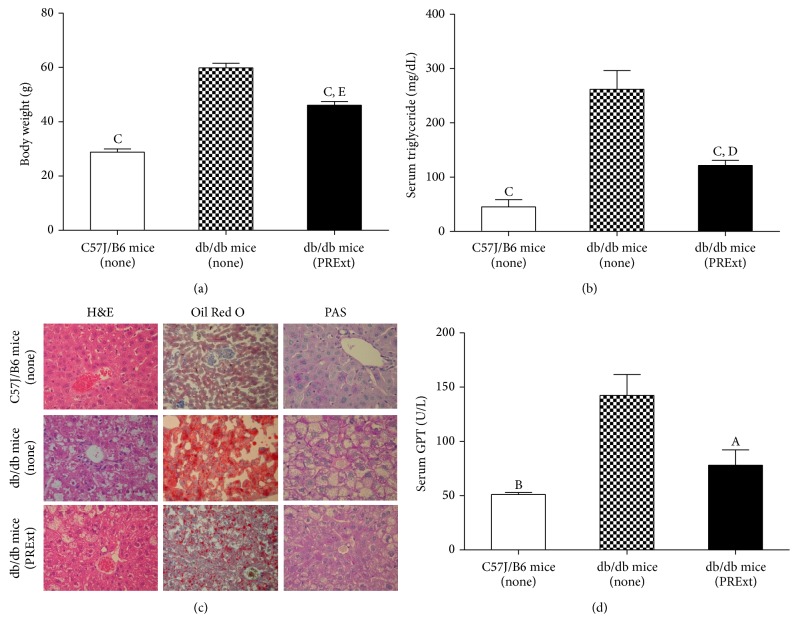
Effects of PRExt administration on the attenuation of obesity and hepatic steatosis in db/db mice. (a) Body weight and (b) serum triglyceride levels in db/db control mice and mice treated with PRExt. (c) Representative results from mouse liver tissue sections stained with H&E, Oil red O (ORO), and PAS stain. (d) Quantification of serum GPT levels in db/db control mice and mice treated with PRExt. Data are presented as the mean ± SEM (*n* = 6–10/group). ^A^ *p* < 0.05, ^B^ *p* < 0.01, and ^C^ *p* < 0.001 when compared to untreated db/db mice. ^D^ *p* < 0.05 and ^E^ *p* < 0.01 when compared to C57J/B6 mice.

Obesity and hepatic steatosis are two common comorbidities observed in db/db mice. In our study, the body mass of untreated db/db mice was twofold that of C57J/B6 mice (*p* < 0.001; Figure 6(a)), while PRExt administration resulted in an approximately 15% decrease in body mass in db/db mice (*p* < 0.001). Despite this decrease, the body mass of PRExt treated db/db mice remained significantly higher than that of C57J/B6 mice (*p* < 0.01). Additionally, serum triacylglyceride levels were increased nearly fivefold in untreated db/db mice compared with C57J/B6 mice (*p* < 0.001; see Figure 6(b)); however, a significant decrease in serum triacylglyceride was observed after PRExt treatment (*p* < 0.001). Serum triacylglyceride levels nevertheless remained significantly higher in PRExt treated db/db than in C57J/B6 mice (*p* < 0.05).

As shown in Figure 6(c), abnormal lipid accumulation in the liver, as judged by H&E staining of liver tissue, was evident in untreated db/db mice, and these results correlated with heavy Oil red O staining from the same tissue sections. Additionally, the loss of PAS staining in the liver tissue of db/db mice further indicated the loss of hepatic glycogen. However, when db/db mice were treated with PRExt, the histological characteristics of nonalcoholic fatty liver disease (NAFLD) in db/db mice appeared to be attenuated, as seen by histological patterns similar to those of liver tissue obtained from C57J/B6 mice.

Liver injury is judged by serum level of glutamic-pyruvate transaminase (GPT). In the current study, serum GPT in untreated db/db mice was significantly elevated compared with that of C57J/B6 mice (*p* < 0.01; see Figure 6(d)). Corresponding with the observed attenuation of fatty liver pathology in db/db mice treated with PRExt, serum GPT also significantly decreased in these mice (*p* < 0.05).

## 4. Discussion

In the current study, we hypothesized that PRExt, an herbal extract comprising many phytochemicals with beneficial antidiabetes effects, could meet the criteria for a hypoglycemic drug/health food. Therefore, through the use of liver, muscle, and pancreatic beta-cell lines, we evaluated the hypoglycemic actions of PRExt and each of the four constituents. Hepatic gluconeogenesis is regulated by hormone-mediated transcription of the rate limiting enzymes including PEPCK [24]. Using H4IIE liver cells, we firstly demonstrated that the suppressive activity of PRExt on dexamethasone and 8-bromo-cAMP induced PEPCK mRNA expression as previously described [5]. Pentagalloylglucose and the tannin-polymers containing fraction were identified as the two constituents responsible for this effect [7].

Additionally, peripheral glucose uptake by muscle tissue accounts for two-thirds of postprandial glucose disposal [25]. In the current study, PRExt increasingly stimulated glucose uptake in differentiated L6 myotubes up to a concentration of 50 *μ*g/mL. Paeoniflorin, pentagalloylglucose, and tannins have been reported to stimulate peripheral glucose uptake via various mechanisms [9, 10, 26–29]. We also have similar observations (data not shown). Because the 2-NDBG and ^18^FDG are both nonmetabolizable tracers, the increment of ^18^FDG content in limbs of db/db mice treated with PRExt also suggested that glucose absorption in the skeletal muscle is enhanced by PRExt treatment.

How to maintain and promote glucose-mediated insulin secretion is one of the major issues for diabetes therapy. Finally, we describe for the first time the insulinotropic effect of PRExt using BRIN-BD11 pancreatic beta cells. Among the four PRExt constituents described here, only pentagalloylglucose and PEF enhanced glucose-stimulated insulin secretion. Considering functional insulin receptors are known to present in pancreatic beta cells and provide important role in regulating glucose-mediated insulin secretion; insulinotropic effect of pentagalloylglucose might result from its insulin mimetic property [29–31]. On the other hand, the underlying mechanism to explain insulinotropic effect of PEF remained to be investigated.

Acute hypoglycemic effect of Radix Paeoniae Rubra ethanol extract on diabetic rodents was shown previously [5]. Our results also clearly demonstrated that PRExt treatment effectively decreases fasting hyperglycemia in a dose-dependent manner. In terms of the long-term therapeutic efficacy, PRExt administration gradually attenuates diabetic hyperglycemia over 30 days and significantly improves the HOMA-index of db/db mice. As a result, the improvement of glucose intolerance may be the combinational result of improved insulin resistance, beta-cell function, and peripheral glucose absorption in PRExt treated db/db mice.

Obesity, hyperlipidemia, and NAFLD are related pathological factors causing insulin resistance and metabolic syndrome [32] as well as an increased risk for type 2 diabetes [33]. In our investigation, a loss of body associated with PRExt treatment was evident in db/db mice. This decrease in body mass was associated with a reduction in fat pads (data not shown), reduction of serum triglyceride levels, and the attenuation of NAFLD-induced hepatic injury. In terms of hepatic pathohistological results, the vacuole morphology of the hepatic cytoplasm in db/db mice after H&E staining was evident as a result of washing out the intracellular triacylglyceride. It is correlated with heavy Oil red O staining from the same tissue and companioned the loss of PAS staining which indicates the loss of hepatic glycogen. Those NAFLD features observed in db/db mice were changed by PRExt administration. However, it should be noted that PRExt treatment was only partially effective, as only differences in body mass and serum triglyceride levels between treated and untreated mice are significant.

The present study indicates that the antidiabetic activity of PRExt is associated with the polyphenols present in PRExt. Polyphenols are well reported to have poor pharmacokinetic properties [34–36]; therefore, they lack drug-ability due to the requirement of high oral dosages. However, our results also pointed out the potential complementary actions of the constituents in PRExt because of the overlapping biological activity from different compounds. In addition, targeting multiple biological activities is also a good strategy for treating multifactorial disease such as diabetes; therefore, it may provide some explanation for the effectiveness of herbal extract with a pool of polyphenols in treating diabetic mice.

In conclusion, the present investigation provides novel evidence of insulinotropic effects of PRExt, pentagalloylglucose, and PEF on insulin secreting cells, BRIN-BD11 cells. Additionally, beneficial antidiabetic effects of the long-term PRExt (200 mg/kg) administration on db/db mice were demonstrated. Our research results and literatures also suggest that paeoniflorin, pentagalloylglucose, and PEF can serve as useful standards with hypoglycemic activities for a quality-controlled herbal extract made from Radix Paeoniae Rubra for the purpose of treating type 2 diabetes.

## Supplementary Material

The LC-MS profile paeoniflorin and PGG, as shown in Suppl. Figs. 1 (positive mode) and 2 (negative mode) by a modified HPLC condition. The peaks at 13.55 min and at 13.71 min in Suppl. Fig. 1C and 2C, respectively, were confirmed to be paeoniflorin. By a similar manner, the peaks at 19.02 min (Suppl. Fig. 1D) and 18.30 min (Suppl. Fig. 2D) were identified as PGG. The MS signal of paeonol was not detected due to its limited content in the extract.

## Abbreviations

FDG: [^18^F] 2-fluorodeoxyglucose
2-NBDG: 2-(N-(7-Nitrobenz-2-oxa-1,3-diazol-4-yl)Amino)-2-deoxyglucose
cps: Counts per second
DM: Diabetes mellitus
DMEM: Dulbecco's Modified Eagle's Medium
EtOH: Ethanol
FBS: Fetal bovine serum
H&E: Hematoxylin and eosin
IVGTT: Intravenous glucose tolerance test
KRBB: Krebs-Ringer Bicarbonate Buffer
NAFLD: Nonalcoholic fatty liver disease
ORO: Oil red O
PEF: Polyphenol-enriched fraction
PAS: Periodic Acid-Schiff
PEPCK: Phosphoenolpyruvate carboxykinase
HOMA: Homeostasis model assessment
HTRF: Homogeneous Time Resolved Fluorescence
HPLC: High Performance Liquid Chromatography
HPSEC: High Pressure Size Exclusion Chromatography
ANOVA: One-way analysis of variance
PET: Positron Emission Tomography
ROI: Region of interest
PRExt: The ethanol extract of Radix Paeoniae Rubra
*λ*max: The Lambda max
TCM: Traditional Chinese Medicine
T2D: Type 2 diabetes.
