# Supplementary material for: Evaluation of the In Vivo Therapeutic Effects of Radix Paeoniae Rubra Ethanol Extract with the Hypoglycemic Activities Measured from Multiple Cell-Based Assays
Source: Evid Based Complement Alternat Med. 2016 Nov 29;2016:3262790. doi: 10.1155/2016/3262790 (PMC5153506; doi:10.1155/2016/3262790)

Supplementary Figure 1. LC-MS profile of PRExt in positive mode.

(A) Full HPLC chromatogram monitored at 254 nm (B) signal-magnified (6.7 ×) HPLC chromatogram monitored at 254 nm (C) MS spectrum of paeoniflorin (*m/z* 503, $[M + Na]^+$) at 13.55 min (D) MS spectrum of pentagalloylglucose (*m/z* 963, $[M + Na]^+$) at 19.02 min. The molecular weight of paeoniflorin and pentagalloylglucose is 480.47 and 940.68, respectively.

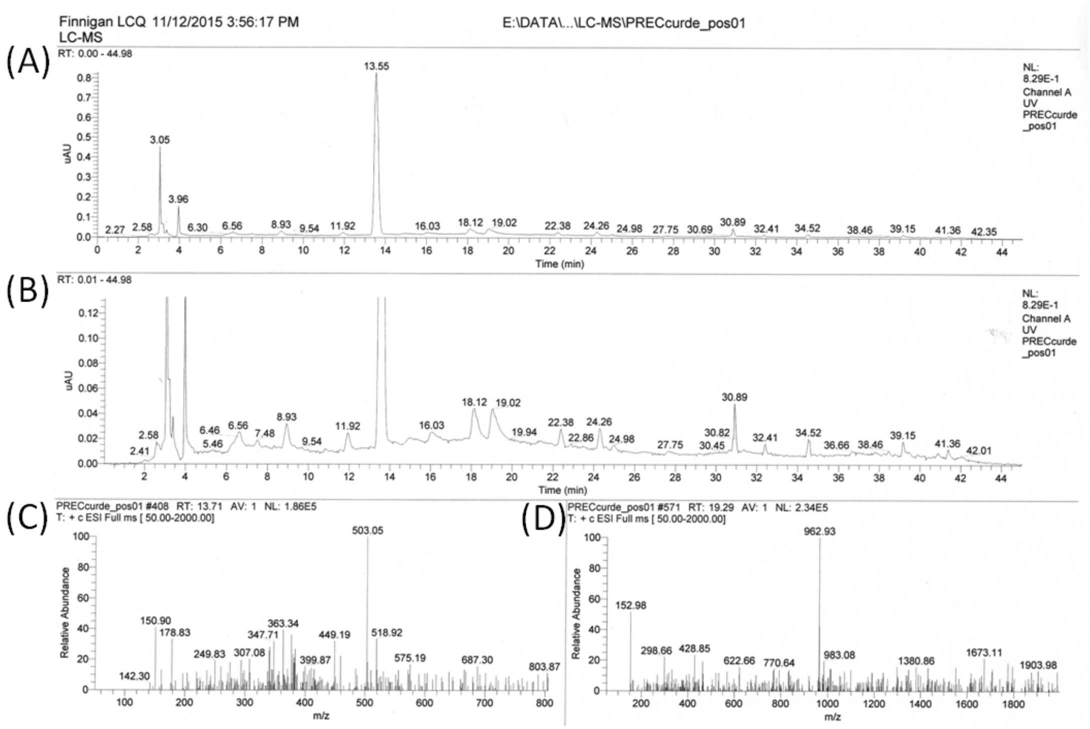

Supplementary Figure 2. LC-MS profile of PRExt in negative mode.

(A) Full HPLC chromatogram monitored at 254 nm (B) signal-magnified (12.5 ×) HPLC chromatogram monitored at 254 nm (C) MS spectrum of paeoniflorin (*m/z* 479, $[M - H]^{-}$) at 13.71 min (D) MS spectrum of pentagalloylglucose (*m/z* 939, $[M + Na]^{+}$) at 18.30 min

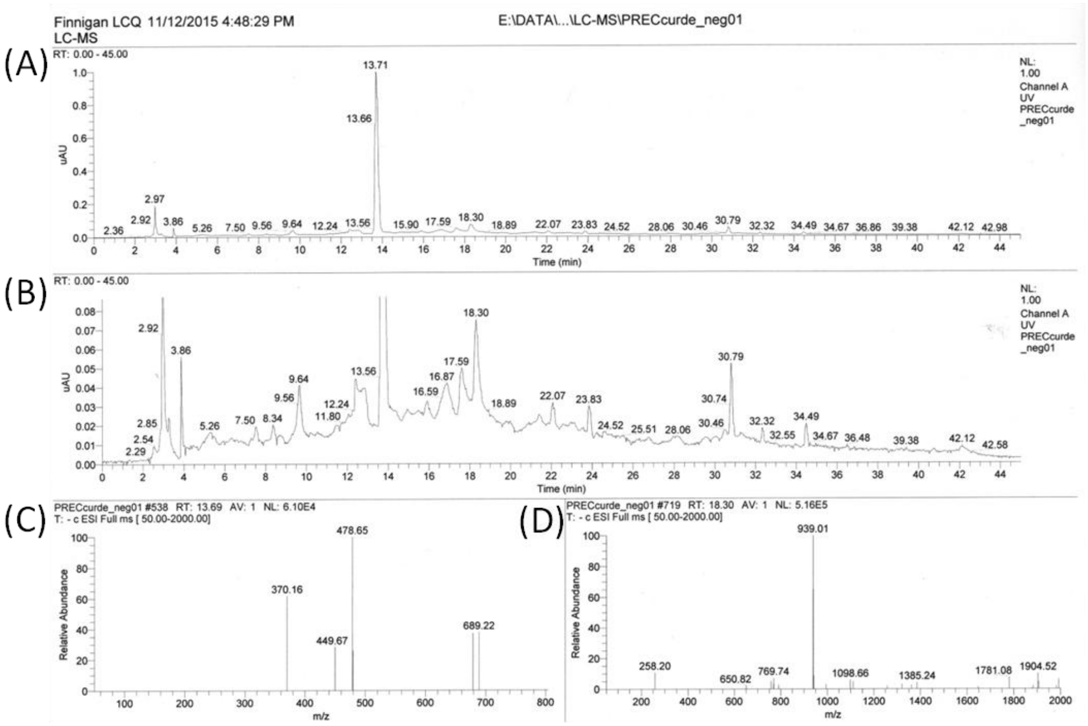

Supplement: Supplementary file 1 — The LC-MS profile paeoniflorin and PGG, as shown in Suppl. Figs. 1 (positive mode) and 2 (negative mode) by a modified HPLC condition. The peaks at 13.55 min and at 13.71 min in Suppl. Fig. 1C and 2C, respectively, were confirmed to be paeoniflorin. By a similar manner, the peaks at 19.02 min (Suppl. Fig. 1D) and 18.30 min (Suppl. Fig. 2D) were identified as PGG. The MS signal of paeonol was not detected due to its limited content in the extract. [file 3262790.f1.pdf]
